# Is Polysialylated NCAM Not Only a Regulator during Brain Development But also during the Formation of Other Organs?

**DOI:** 10.3390/biology6020027

**Published:** 2017-04-27

**Authors:** Christina E. Galuska, Thomas Lütteke, Sebastian P. Galuska

**Affiliations:** 1 Department of Reproductive Biology, Leibniz Institute for Farm Animal Biology (FBN), Wilhelm-Stahl-Allee 2, 18196 Dummerstorf, Germany; galuska.christina@fbn-dummerstorf.de; 2 ITech Progress GmbH, Donnersbergweg 4, 67059 Ludwigshafen, Germany; thomas.luetteke@vetmed.uni-giessen.de

**Keywords:** polysialic acid, NCAM, sialic acids, cell adhesion molecule, organogenesis, pre- and postnatal development

## Abstract

In mammals several cell adhesion molecules are involved during the pre- and postnatal development of all organ systems. A very prominent member of this family is the neural cell adhesion molecule (NCAM). Interestingly, NCAM can be a target for a special form of posttranslational modification: polysialylation. Whereas nearly all extracellular proteins bear mono-sialic acid residues, only a very small group can be polysialylated. Polysialic acid is a highly negatively-charged sugar polymer and can comprise more than 90 sialic acid residues in postnatal mouse brains increasing dramatically the hydrodynamic radius of their carriers. Thus, adhesion and communication processes on cell surfaces are strongly influenced allowing, e.g., the migration of neuronal progenitor cells. In the developing brain the essential role of polysialylated NCAM has been demonstrated in many studies. In comparison to the neuronal system, however, during the formation of other organs the impact of the polysialylated form of NCAM is not well characterized and the number of studies is limited so far. This review summarizes these observations and discusses possible roles of polysialylated NCAM during the development of organs other than the brain.

## 1. Introduction

**Figure 1:**
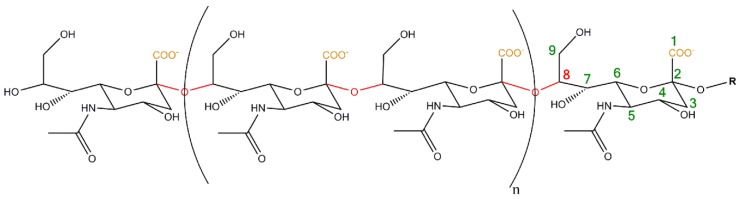
Chemical structure of polysialic acid (polySia): In mammals polySia consists of α2,8-linked *N*-acetylneuraminic acid residues (Neu5Ac) (linkage in red). Neu5Ac belongs to the wider family of sialic acids [15,16,17]. It is an α-keto acid with a nine carbon backbone (numbering in green) bearing a carboxylate anion under physiological conditions (orange). R: N-glycan or O-glycan.

Sialic acid polymers (Figure 1) are frequently present in nature ranging from echinoderms to mammals [1,2,3,4,5]. However, distinct bacteria strains, like *Escherichia coli* (*E. coli*) K1, are also able to generate different polysialic acid (polySia) species [1,6,7]. In the brain of mammals polySia is mainly present on the neural cell adhesion molecule (NCAM) [8]. Based on the first detection of the polysialylated form of NCAM in the brain by Jukka Finne 35 years ago [9,10], numerous studies characterized the various biological functions of polysialylated NCAM in the brain of vertebrates depending among other things on the stage of development [5,11,12,13]. Not least through the observation of many substantial defects during brain development in polySia knock-out mice by Rita Gerardy-Schahn’s lab and cooperation partners, the essential impact of this posttranslational modification was demonstrated for the neuronal system [14]. However, the additional lethal phenotype of polySia knock-out mice may not only be the result of the dramatic changes in the brain, since polysialylated NCAM is also present in several other essential organs during organogenesis like the developing heart, kidney and liver.

This review recaps selected discoveries regarding the polysialylated forms of NCAM outside the neuronal system, and starts with an overview concerning the polysialylation process of NCAM and the biochemical impact of polySia on NCAM.

## 2. Polysialylation of NCAM

**Figure 2:**
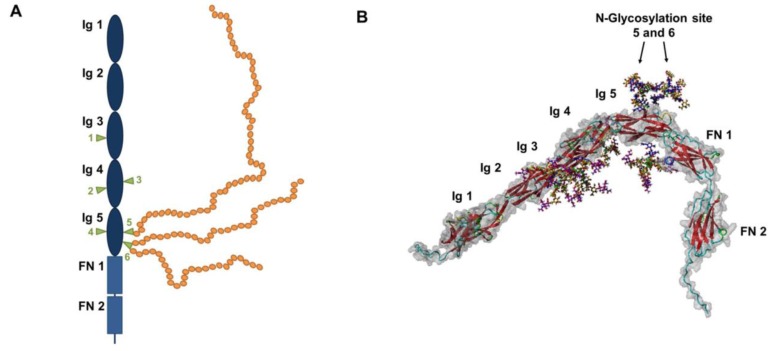
Models of NCAM and polySia: (**A**) All three major isoforms of NCAM consist of two fibronectin (FN) and five immunoglobulin like (Ig) domains, and six N-glycosylation sites were characterized (green triangles) [18]. PolySia (orange balls) can be present on N-glycans of glycosylation sites 5 and 6. (**B**) The 3D model of NCAM was created as described in Ulm et al. [22]. The structure of the NCAM was created with the homology modeling software Modeler [23]. Template search in the Protein Data Bank [24] as well as the creation of sequence alignments was performed with the BLAST service [25]. Carbon atoms of the glycan chains are colored by residue types using the color scheme of the symbol nomenclature for glycans (SNFG) [26].

Three major isoforms of NCAM are expressed and can be polysialylated in mammals; NCAM-180, NCAM-140, and NCAM-120 [18,19,20,21]. NCAM-180 and NCAM-140 are transmembrane proteins, whereas NCAM-120 is a glycosyl-phosphatidylinositol (GPI)-anchored protein. All three isoforms contain five immunoglobulin (Ig)-like domains and two fibronectin (FN) domains (Figure 2).

**Figure 3:**
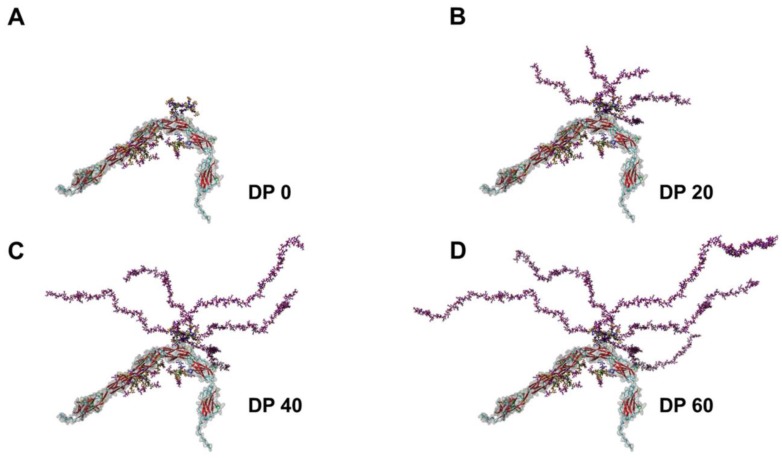
3D models of polysialylated NCAM: Four different polysialylation stages are depicted: (**A**) no polySia; (**B**) DP 20; (**C**) DP 40; and (**D**) DP 60. The polySia chains of the glycan models were created as described earlier [22].

Six N-glycosylation sites have been described. The polysialylation of the protein backbone typically takes place on N-glycans at glycosylation sites 5 and 6 of the 5th Ig-domain in vivo [22,23,24,25,26]. In postnatal mouse brains, the majority of these N-glycans bear two or more polySia chains and polymers with more than 90 sialic acid residues could be detected [27]. Remarkably, between 60 and 90 *N*-acetylneuraminic acid (Neu5Ac) residues seem to be present on the largest part of N-glycans. As illustrated in Figure 3, already with a degree of polymerization (DP) of 40 sialic acid residues polySia chains represent the dominating element of NCAM.

Two polysialyltransferases were described to modify NCAM; ST8SiaII and ST8SiaIV [28,29]. Interestingly, in vitro the polysialyltransferases can also polysialylate themselves [30,31]. However, so far only the polysialylated form of ST8SiaII has been detected in vivo [32]. In addition to NCAM and the polysialyltransferases six further polySia-carriers were identified in mammals:

- A sodium channel in adult rat brain [33];
- Cluster of differentiation (CD) 36 in murine and human milk [34];
- Neuropilin-2 on dendritic cells, macrophages and microglia (mouse and human) [22,35,36,37];
- C-C chemokine receptor type 7 (CCR7) on dendritic cells (mouse and human) [38];
- Synaptic cell adhesion molecule SynCAM-1 on polydendrocytes (NG2) cells in postnatal mouse brain [39]; and
- E-selectin ligand-1 on microglia and macrophages (mouse and human) [40].

Whereas the mechanisms of protein specific polysialylation for these six polySia carriers are more or less unknown, several studies characterized the polysialylation process of NCAM [2,41].

Twenty years ago the minimal structure of NCAM was determined by Nelson and colleagues, which is needed for an efficient polysialylation [42]. Additionally, the place of polysialylation, the 5th Ig-domain, the 4th Ig-domain, as well as the first FN domain were found to be necessary. Eight years later Colley and Co-workers demonstrated that also a truncated form without the 4th Ig-domain represent an adequate acceptor structure [43].

During the last 10 years especially the team of Karen Colley follows the idea that a protein-protein interaction between NCAM and the polysialyltransferases could be essential and they were able to define the structural requirements in more detail. Using a charge distribution analysis, an acidic patch was recognized in the first FN-domain, which is formed by Asp^520^, Glu^521^, and Glu^523^ [44]. Furthermore, the three positively-charged amino acids were not only shown to be important for the polysialylation capacity, but also for an initial binding of the polysialyltransferase ST8SiaIV [45]. In accordance with the acidic patch of the first FN-domain in the case of NCAM, europilin-2 also exhibits an acidic area located in the MAM domain, which was shown to be essential for polysialylation by ST8SiaIV [46]. Thus, distinct negatively-charged areas of the acceptor protein might be generally important for the initiation of polysialylation.

**Figure 4:**
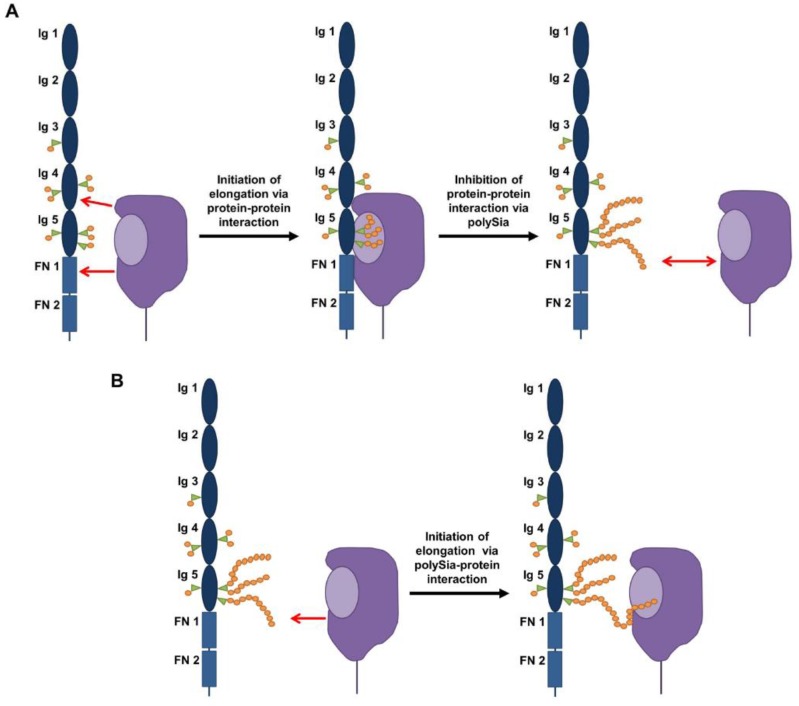
Proposed polysialylation mechanism based on [2,29,41,47,48]. (**A**) To start polysialylation, polysialyltransferases interact with areas of the 4th Ig- and the first FN-domain [2,41]. In addition, a terminal sialylation must be present [49]. After the initiation phase polysialylation starts. (**B**) Since the polymers are negatively charged and the chain length continuously increases, it was proposed that the polysialyltransferases switch from a protein-protein interaction to a glycan-protein interaction to continue polySia synthesis [47].

Interestingly, polysialyltransferases comprise basic regions suggesting that an enzyme acceptor complex is initiated by electrostatic attraction with the acidic regions of NCAM to start polysialylation of NCAM (illustrated in Figure 4A) [47,48]. Troy and co-workers described a polysialyltransferase domain (PSTD) localized close to the sialylmotif-S (SM-S) consisting of 32 amino acids. This basic patch is necessary for polysialylation of NCAM. In the case of ST8SiaIV, the amino acids Ile^275^, Lys^276^, Arg^272^, and Arg^252^ seem to be particularly important. Moreover, this basic region is discussed to switch from a protein-protein interaction between NCAM and ST8SiaIV to a protein-carbohydrate interaction with the nascent polySia chain during the polysialylation process (Figure 4B) [47].

Further amino acids modulating the recognition of NCAM were identified when in Karen Colley’s labs the amino acid sequences were examined for positively-charged areas. They identified a polybasic region (PBR) between the amino acids 86–120 and 71–105 in ST8SiaII and ST8SiaIV, respectively [50]. Arg^82^ and Arg^93^ are discussed to play a special role during interaction and polysialylation process [51]. Very recently, the team could be verified by nuclear magnetic resonance (NMR) analysis that the acidic patch of the FN-domain interacts with the outlined basic region of ST8SiaIV [48]. All of the studies are focused on ST8SiaIV, but it is likely that similar mechanisms take place during the polysialylation by ST8SiaII. Intriguingly, Arg^82^ is also involved in the recognition and/or binding phase during the polysialylation of neuropilin-2 and SynCAM-1 [51].

The crystal structure of ST8SiaIII, which is discussed to build sialic acid oligomers [4], support the models of Frederic Troy and Karen Colley [52]. Strynadka and colleagues compared—on the basis of their crystal structure—the amino acid sequences of ST8SiaII and IV with ST8SiaIII. The generated 3D model of ST8SiaIV nicely visualizes the potential interaction areas between ST8SiaIV and NCAM. PSTD seems to be involved in binding and coordinating of the glycan acceptors forming a positively-charged stretch to the active site. Interestingly, PBR, as well as PSTD, seem to initiate the interaction between the polysialyltransferase and the acidic patch of the first FN-domain. Thereby, Arg^93^ of ST8SiaIV forms a salt bridge with Asp^521^.

Nevertheless, many questions have still to be answered concerning the “specific” recognition and polysialylation mechanisms by ST8SiaII and ST8SiaIV. For example, are all of these acceptor proteinsin acidic patches present? In addition, the elongation process is still mysterious, since the sialic acid polymers can be longer than the complete protein backbone of NCAM [22]. Moreover, N-glycans of both glycosylation sites (5th and 6th) are potential acceptors and the present N-glycans can be modified with more than one polySia chain. How can the polysialyltransferases handle this situation? Is a switch between protein-protein and protein-glycan interaction (Figure 4), as well as a parallel switch between nascent chains on glycosylation site 5 and 6, possible during the elongation phase, and what is the exact mechanism? It will be interesting to see how understanding of these issues will evolve in the coming years.

## 3. Impact of PolySia

The dimension and number of polySia chains present on NCAM already suggest that the function of NCAM is strongly modulated by these linear but very flexible carbohydrate chains (Figure 3) [22]. However, polySia does not only modulate NCAM-dependent mechanisms, but can also influence numerous processes by itself [2].

Nevertheless, the most prominent example is still the tuning of cell-cell adhesion mechanism by the inhibition of the homophilic NCAM-NCAM interaction. More than twenty-five years ago Rutishauser and co-workers proposed that the cell-cell interaction is triggered depending on the polySia ratio of NCAM [53,54]. While unpolysialylated NCAM manifests cell adhesion via homophilic binding in *trans* mode, increasing amounts of polySia abolish the interaction between NCAM molecules expanding the area between cells. Due to the dramatic increase of the hydrodynamic radius also cell-cell interactions mediated by other adhesion molecules like cadherins can also be negatively affected [55]. Thus, polySia represents a strong regulator of cell-cell interactions, as well as communication processes. In the developing brain the loss of polySia has enormous consequences. For instance, the enzymatic degradation of polySia on olfactory precursors leads to an inhibition of their migration capacity, and fewer cells reach the bulbus olfactorius [56,57].

As already mentioned, polySia can regulate physiological processes independently of its carrier. Especially Sato’s and Kitajima’s groups identified several biomolecules, which bind polySia inducing or inhibiting distinct signaling pathways [2,58]. Interestingly, some of these interactions seem to require a minimum degree of polymerization (DP). For example, brain-derived neurotrophic factor (BDNF) can only bind polySia chains consisting of more than eleven sialic acid residues [59]. Based on their finding, Sato and co-workers proposed that these interactions lead to an accumulation of BDNF on the cell surface resulting in a reservoir of the neurotrophin BDNF. Since the affinity between BDNF and its receptors is higher than between polySia and BDNF, it seems to be possible that BDNF molecules can continuously switch from the polySia-BDNF-reservoir to their receptors TrkB and p75NTR. Comparable results were obtained, when nerve growth factor (NGF), neurotrophin-3 (NT-3), as well as neurotrophin-4 (NT-4) were analyzed [59]. Interestingly, they also show that the BDNF-polySia complex leads to an up-regulation of growth or/and survival of neuroblastoma cells. Their results are in line with previous findings demonstrating a connection between the formation of BDNF-polySia complexes and the survival of neurons [60].

Moreover, fibroblast growth factor 2 (FGF2) was shown to be an interaction partner of polySia [61]. The complex formation requires a minimum chain length of 17 sialic acid units. Intriguingly, polySia mediates FGF2 signaling in a negative way leading to an inhibition of FGF2-stimulated cell growth. It seems to be that in contrast to BDNF, FGF2 cannot be directly transferred to its receptor and a previous migration to heparin sulfate is necessary, before FGF2 can be recognized by fibroblast growth factor receptors (FGFR).

In addition to the presented modulation of NCAM functions as well as interaction partners, some others functionalities of polySia were described (excellently reviewed in [2,5,11,13,18,56,58,62]). However, the addressed examples represent the main roles of polySia which, so far, have been discussed to take place during the development of other organs than the brain.

## 4. PolySia-NCAM during the Development of the Liver

**Figure 5:**
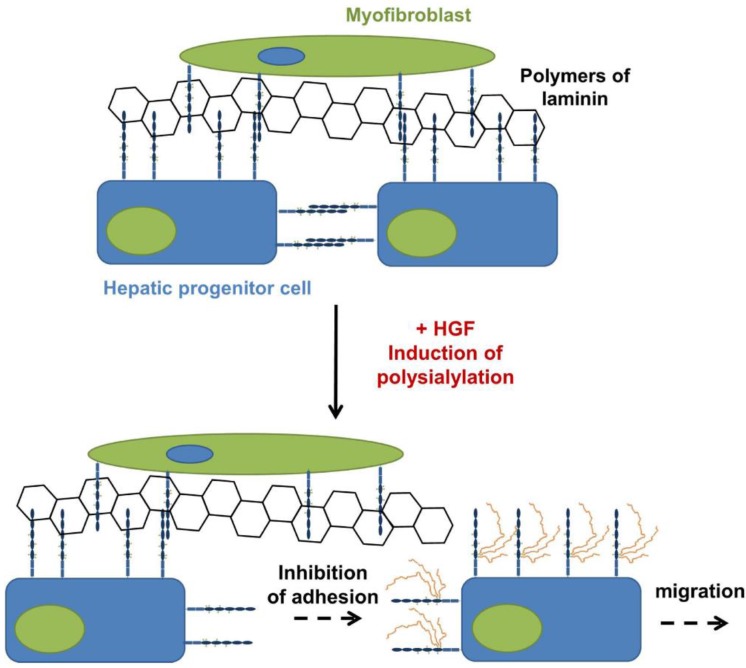
PolySia modulates the migration of hepatic progenitor cells: Inactive hepatic progenitor cells express unpolysialylated NCAM [64]. Hepatic progenitor cells interact with myofibroblasts and laminin via NCAM. After activation by hepatic growth factor (HGF) polysialylation is induced, allowing a migration of activated hepatic progenitor cells.

The liver is the central organ for metabolism and the biggest gland in vertebrates playing an essential role in physiological balance. Already in the 1990s polySia was detected during prenatal development of the liver [63]. Whereas hepatocytes and liver parenchyma showed no polySia signal, interstitial areas were polySia positive during liver organogenesis. Later, Forbes and co-workers observed that murine hepatic progenitor cells express polysialylated NCAM and that during differentiation to hepatocytes the expression levels decrease [64]. Using cell based assays they demonstrated that polySia inhibits the cell matrix interaction, counteracts cell aggregation and increases hepatocyte growth factor-induced migration of hepatic progenitor cells (Figure 5). Furthermore, polySia weakens the interaction with NCAM-positive myofibroblasts.

Interestingly, in postnatal polySia knockout mice, impaired bile duct structures were observed. Moreover, polySia is involved during regeneration after liver injury. During liver injury the ductular reaction is initiated leading among other things to an increasing number of ducts associated with matrix production, migration and proliferation of progenitor cells, as well as subsequent differentiation into hepatocytes [65]. This reaction comes along with an upregulation of polySia on the cell surface of hepatic progenitor cells. Intriguingly, enzymatic removal of sialic acid polymers inhibits the ductular reaction since the cell-cell and cell-matrix adhesion can no longer be fine-tuned by polySia [64]. Thus, polysialylated NCAM represent a key element during organogenesis, as well as regeneration also in the liver.

## 5. PolySia-NCAM during the Development of the Heart

During the 1990s polysialylation status was examined in rat and chicken hearts by Western blotting against polySia and NCAM using several pre- to postnatal and adult stages [66,67]. It seems that the concentration of polysialylated NCAM increases during prenatal development, whereas during postnatal development the expression levels decrease. No polySia was detectable in adult samples and only small amounts of unpolysialylated NCAM were stained. Additionally, immunohistochemistry was performed to localize polySia generation using tissue slides. In rat, as well as in chicken, samples myocardial cells were polySia-positive. The authors suggested that due to the anti-adhesive properties, polySia takes part during the modeling of the myocardium representing the muscle tissue of the heart.

Furthermore, the epicardial layer, consisting mainly of connective tissue, exhibited polySia signals. Additionally, in areas of migrating cells, forming the mesenchyme, polySia was present suggesting that also here the migration capacity of the cells is modulated by polySia. Finally, the innervation of the heart is characterized by high levels of polySia [66]. Since myocardial cells, as well as the areas of neuronal areas, revealed polySia staining, the invasive growth of neuronal connections, as well as the subsequent formation of muscle structure seem to be polySia-dependent events [66]. The impact of polySia, however, cannot be determined, because no data were published so far using a polySia knockout system or other possibilities to prevent a polysialylation during heart development.

## 6. PolySia-NCAM during the Development of the Kidney

The kidney, as a further essential organ of vertebrates, exhibits distinct regions of polySia-NCAM-positive cells during organogenesis in rats [68]. In adult kidneys polySia is no longer synthesized. Primarily early structures, like the ureteric bud—later building the collecting duct system, which connects the nephrons and the ureter—in addition to the metanephrogenic mesenchyme—forming after conversion to epithelium cells the nephrons—are polySia-NCAM-positive. Additionally, after the onset of nephrogenesis polySia is still present. Roth and co-workers suggested that polySia may support, via its anti-adhesive properties and modulation of cell-cell interaction, the assembly of the complex structure of the nephron. The nephron is the functional unit of the kidney and, thereby, regulating the concentration of water and soluble substances. An impaired formation of the tight junction between the epithelial cells of the nephron during organogenesis would lead to disturbance of renal function. Animal studies elucidating the exact role of polySia during kidney formation and the consequences of a loss of polySia have not yet been published.

## 7. PolySia-NCAM during the Development of the Testis

The testis contains mainly seminiferous tubules consisting of developing germ cells (spermatogonia, spermatocytes, spermatids, and spermatozoon). In addition, interstitial cells are located between the tubules. In addition to interstitial macrophages, Leydig cells are primarily present in the interstitium. Leydig cells belong to the endocrine system and they release, inter alia, testosterone and other androgens, such as androstenedione and dehydroepiandrosterone (DHEA). Thus, Leydig cells are important regulators for sexual development and spermatogenesis. Mayerhofer et al. detected NCAM on Leydig cells of adult testes in the beginning of the 1990s [69]. Shortly thereafter they also observed that, during development of murine testicular Leydig cells, the polysialylated form of NCAM is also present [70]. Intriguingly, clustered Leydig cells show stronger polySia staining than isolated Leydig cells and the authors proposed that besides the involvement during the migration into the developing testis, cytodifferentiation and/or cluster formation of Leydig cells is also controlled by polySia and NCAM [69].

Not only interstitial cells, but also Sertoli cells and germ cells, seem to express the polysialylated form of NCAM during fetal development. In feline (fetal samples) and murine (postnatal day 1) testes, membranes of Sertoli cells and/or spermatogonia are polySia-positive [71].

Since neurotrophins like BDNF, NGF, and NT-3 are discussed to support the forming of the seminiferous cord, as well as the persistence of germ cells [72], and polySia directly interacts with these biomolecules influencing their mode of action [2,58,73], the authors propose that polySia might have a direct impact during seminiferous tubule development and initiation of spermatogenesis [74].

This possibility is supported by findings using the roe deer as a model for seasonal initiation and termination of spermatogenesis [74]. In wildlife the mating season is often a restricted period [75]. Thus, spermatogenesis is not necessary during the whole year. For instance, in roe deer, during winter, the seminiferous tubule consists mainly of spermatogonia and Sertoli cells representing the “governess” of germ cells [76,77]. In spring, however, proliferation of spermatogonia rapidly increases and the first spermatocytes and spermatids can be present. During the next weeks the germinal epithelium reaches complete functionality. Already in August the activity of spermatogenesis decreases. Intriguingly, polysialylated NCAM is mainly present during the onset of spermatogenesis (April), as well as when a complete offset occurs (December) [74]. Mainly spermatogonia and Sertoli cells showed polySia staining during these periods. In April, spermatocytes are also present in polySia-positive areas. However, it seems that the signal belongs to Sertoli cells and not to the first wave of spermatocytes.

**Figure 6:**
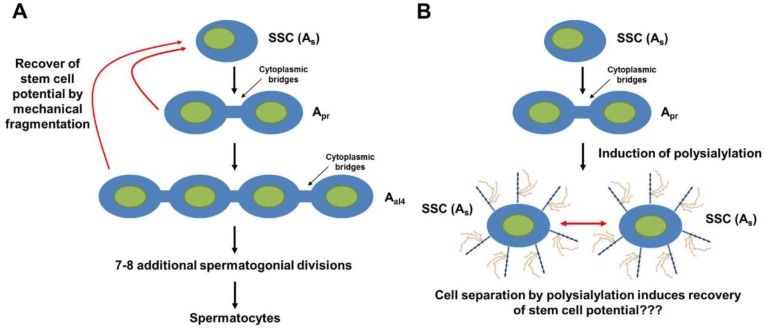
Is a recovery of stem cell potential by polysialylation possible? (**A**) Spermatogonia stem cells (SSC) start to differentiate [80]. The daughter cells are connected via cytoplasmic bridges. A mechanical fragmentation of the connected daughter cells leads to a recovery of stem cell potential [78,79]. (**B**) It was speculated that polysialylation may support a destruction of cytoplasmic bridges and the separation of daughter cells [74]. So far, however, no direct experimental evidence exists.

The initiation of polysialylation during these key points of seasonal spermatogenesis and the ability of polySia to modulate cell-cell interaction and communication, as well as the functionality of growth factor, may contribute to the regulatory system of spermatogenesis. A study showing that spermatogonia differentiation is reversible and stem cell potential is regained when their connections are detached [78,79] let us speculate that polySia might be able to support such a recovery event (Figure 6). However, as of yet, no functional assays were applied to test this hypothesis.

## 8. PolySia-NCAM during the Development of the Epididymis

During epididymal transit sperm matures. This maturation step includes an exchange of several surface components between epithelial cells and sperm cells, which is essential for them to become fertile [81]. Interestingly, it seems to be that the secretion of polysialylated NCAM, as well as polysialylated ST8SiaII by epithelial cells, represents a part of this maturation stage [32].

**Figure 7:**
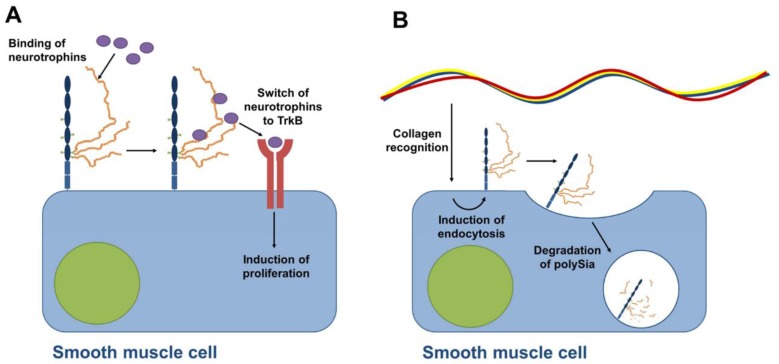
Proposed models of polySia-dependent mechanisms on smooth muscle cells during postnatal development of epididymis [71]. (**A**) The model is based on studies by Sato and co-workers demonstrating that polySia binds neurotrophins modulating the induction of proliferation [58]. Since polySia was observed on proliferating smooth muscle cells and smooth muscle cells express neurotrophin receptors during postnatal development [72] it was suggested that polySia contributes to the induction of proliferation [71]. (**B**) With increasing amounts of collagen the polysialylation of smooth muscle cells stops. Based on cell culture experiments in the lab of Curtis showing that collagen induces the internalization of polySia-NCAM and degradation of polySia chains [83], a functional relationship between increasing amount of collagen and decreasing amount of polySia-NCAM was discussed [71].

Nevertheless, also during the postnatal development of murine epididymis polysialylated NCAM is expressed [71]. In addition to scattered epithelial cells in all areas of the epididymis, primarily proliferating smooth muscle cells exhibit strong polySia staining directly after birth. Intriguingly, comparable results were also obtained, when the postnatal oviduct was examined [71]. In the epididymis during the first ten days after birth the amount of polysialylated NCAM decreases slightly, whereas a dramatic drop down was observed thereafter. On postnatal day 25, for example, no polySia-positive cells, and also no proliferating smooth muscles cells, were detectable. Similar to testis development, during epididymis development neurotrophins and their receptors were also described, which were mainly present in areas of α smooth muscle actin (SMA) positive cells [72]. Taken together, polySia might also modulate the proliferation and/or differentiation of smooth muscle via interactions with neurotrophins as described for neuronal cells [58,71] (illustrated in Figure 7A).

Remarkably, the reduction of the polySia levels comes along with increasing quantities of extracellular collagen during the formation of these contractile areas. A comparable interrelation was also observed in the postnatal tunica albuginea [71], which enclose the testis and consists of fibroblast, myofibroblast and smooth muscle cells and extracellular matrix components like collagen [82]. An in cellulo study by Curtis and co-workers described a possible explanation for the contrarily-regulated amounts of polysialylated NCAM and collagen [83]. They observed an internalization and desialylation of polySia-NCAM, specifically triggered by extracellular collagen. Thus, polySia might play a role during the establishment of contractile arrangements between smooth muscle cells in developing organs and may represent a modulator of proliferation and/or differentiation (Figure 7B).

## 9. PolySia during the Development of the Placenta

In mammals the placenta is essential for the development of new life. Interestingly, during pregnancy in humans the placenta consists of embryonic, as well as maternal, cells. This is possible, since embryonic cells invade the uterus leading to the crucial formation of a nutrient/waste exchange system [84]. Thereby, trophoblasts initiate the invasion and are also important to maintain the connection between the mother and her child [85]. Whereas progenitor cytotrophoblasts form together with syncytiotrophoblasts, the villi—a branching system and the functional unit of the placenta—are invasive cytotrophoblasts that invade the uterus to form an “anchor”. Furthermore, invasive cytotrophoblasts support tissue remodeling events in the uterus to increase the perfusion of the system. In humans all three cell types express polySia early in pregnancy [86]. So far, however, the carrier is unknown.

Remarkably, at term nearly no polysialylation occurs. Fisher and co-workers performed in addition functional assays to get an idea, which processes may depend on the polysialylation status of invasive cytotrophoblasts. By an enzymatic removal of polySia they demonstrated in cell culture models that the migration, as well as invasion capacity of invasive cytotrophoblasts, are significantly impaired [86].

Furthermore, it seems that cancer cells of gestational trophoblastic disease tumors overexpress polySia [86]. The occurrence of polySia on cancer cells was also observed on several other tumors like neuroblastoma [87,88,89,90,91]. Thus, polySia might not only represent an essential part during pregnancy, but also an important regulator during placental pathologies.

## 10. Conclusions

Especially due to pioneering work of the lab of Jürgen Roth and cooperation partners, we have known for many years that the polysialylated form of NCAM is not only restricted to the neuronal system during pre- and postnatal development in mammals. In addition to the outlined organs, the development of other physiological systems seems to be supported by polySia-NCAM. For instance, during hair follicle formation, as well as during the development of the digestive and the respiratory elements, the polysialylated form of NCAM is present, as shown in Lackie et al. [63]. Furthermore, polysialylated NCAM in hair cells was discussed to modulate the connection between nerves and sensory cells during cochlea development in mice [92].

Taken together, the described localization, as well as the abilities of polySia to influence cell-cell interaction/communication and proliferation, in addition to differentiation processes, seems to trigger the maturation of organs throughout the body. Nevertheless, organ and/or cell specific ablation of polySia in murine systems will be necessary to determine exactly the various functions of polySia as a posttranslational modification of NCAM in all different physiological systems.
